# Association between the oral microbiota and hyperlipidemia: evidence from a national cross-sectional study

**DOI:** 10.1097/MS9.0000000000005045

**Published:** 2026-04-28

**Authors:** Xiaolin Wu, Shanglin Cai, Zirui Bai, Peiping Li, Yingbin Jia, Jian Li

**Affiliations:** a Department of Hepatobiliary Surgery, The Fifth Affiliated Hospital, Sun Yat-sen University, Zhuhai, P. R. China; b Department of Urology Surgery, The Fifth Affiliated Hospital, Sun Yat-sen University, Zhuhai, P. R. China

**Keywords:** hyperlipidemia, lipid metabolism, NHANES, oral microbiome, Parvimonas, Prevotella

## Abstract

**Background:**

Oral and gut microbiota interact in the pathogenesis of metabolic diseases. However, the associations between the oral microbiome and host lipid metabolism remain unclear. This study aimed to explore the relationship between metabolic syndrome and the oral microbiome.

**Methods:**

Participants from the 2009–2012 National Health and Nutrition Examination Survey database were analyzed. Correlations between alpha diversity and hyperlipidemia, as well as blood lipid levels, were examined. Principal coordinate analysis and permutational multivariate analysis of variance were used to determine differences in microbial composition between groups. Linear discriminant analysis effect size (LEfSe) analysis identified key microbial taxa associated with hyperlipidemia. Cox regression and Kaplan‒Meier methods were applied for survival analyses. Functional Annotation of Prokaryotic Taxa and mediation analyses were used to explore the role of microbial functions in microbiome-mediated hyperlipidemia risk.

**Results:**

A total of 3104 participants were included, with 2215 diagnosed with hyperlipidemia. Multivariate linear regression revealed significant correlations between alpha diversity and total cholesterol and low-density lipoprotein levels (*P* < 0.017). Cox regression indicated that higher oral microbial alpha diversity was associated with a lower risk of cardiovascular mortality (*P* < 0.017). Beta diversity analysis revealed distinct oral microbial profiles between hyperlipidemic and non-hyperlipidemic individuals (*P* < 0.017). LEfSe analysis identified *Prevotella* and *Parvimonas* as key genera enriched in the oral microbiota of hyperlipidemic participants. Sulfur metabolism partially mediated the association between *Parvimonas* and hyperlipidemia.

**Conclusion:**

The oral microbiota is closely associated with host lipid metabolism. *Prevotella* and *Parvimonas* exhibit higher oral abundances in hyperlipidemic individuals, with *Parvimonas* abundance directly correlated with blood lipids. *Parvimonas* may increase hyperlipidemia risk via sulfur metabolism. Further studies are needed to elucidate the underlying mechanisms, which could serve as effective targets for hyperlipidemia management.

## Introduction

Hyperlipidemia is a metabolic disorder characterized by abnormal blood lipid levels, including elevated total cholesterol (TCHO), triglyceride (TG), and low-density lipoprotein cholesterol (LDL-C) or reduced high-density lipoprotein cholesterol (HDL-C)^[^1^]^. In the United States, the prevalence of hyperlipidemia among adults is 53%^[^2^]^. Accumulating evidence indicates that hyperlipidemia is a critical risk factor for cardiovascular morbidity and mortality^[^3–5^]^. However, despite standard interventions, fewer than 35% of patients achieve adequate control^[^6^]^. Thus, a deeper understanding of hyperlipidemia is warranted to develop additional therapeutic strategies.

HIGHLIGHTS

- The study first systematically analyzed the relationship between oral microbiota and hyperlipidemia.
- Alpha diversity of the oral microbiota is positively correlated with blood low-density lipoprotein and total cholesterol, and exhibits an inverted U-shaped nonlinear association with blood high-density lipoprotein.
- *Parvimonas* and *Prevotella* are characteristic genera in the oral microbiota of individuals with hyperlipidemia.
- Sulfur metabolism partially mediated the association between *Parvimonas* and hyperlipidemia.

The gut microbiota is closely associated with host lipid metabolism and hyperlipidemia risk^[^7^]^, and strategies targeting the gut microbiota to reduce lipid levels have been explored^[^8–11^]^. The oral cavity, connected to the large intestine via the digestive tract, is a primary microbial habitat in the upper gastrointestinal tract and is the second most abundant microbial community in the human body^[^12^]^. The oral and gut microbiomes interact and coordinately regulate physiological functions and pathological processes^[^13^]^. Emerging studies have linked the oral microbiome to various cardiovascular and metabolic diseases^[^14,15^]^, including hypertension^[^16^]^, atherosclerosis^[^17^]^, diabetes^[^18^]^, and chronic obstructive pulmonary disease (COPD)^[^19^]^. Intervention in the oral microbiome represents an emerging therapeutic strategy for cardiovascular and metabolic diseases^[^20^]^. Therefore, investigating the association between the oral microbiota and lipid metabolism is clinically relevant. However, no systematic analysis has yet clarified the relationship between the oral microbiota and host lipid metabolism.

This study aimed to explore the association between the oral microbiome and hyperlipidemia using a large national cohort, enhance the understanding of the potential role of the oral microbiome in hyperlipidemia prevention and treatment, and identify potential therapeutic targets.

## Methods

### Study population

The National Health and Nutrition Examination Survey (NHANES) is conducted by the National Center for Health Statistics (NCHS) and consists of a series of cross-sectional, multistage probability surveys of the U.S. population. The data are publicly available on the website of the U.S. Centers for Disease Control and Prevention (CDC) (http://www.cdc.gov/nchs/nhanes.htm). The study protocol was approved by the Research Ethics Review Board of the NCHS. All participants provided written informed consent at enrollment. This cross-sectional study has been reported in line with the STROCSS guidelines^[^21^]^.

**Figure 1.**
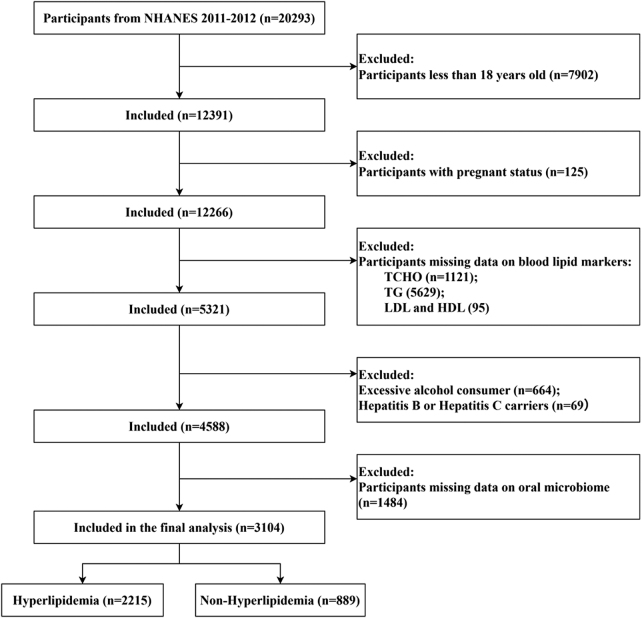
Selection flowcharts.

In the present study, data from all participants in the 2009–2012 NHANES cycles were initially included (*n* = 20 293). The participants were subsequently excluded on the basis of the following criteria: (1) aged <18 years (*n* = 7902); (2) pregnant (*n* = 125); (3) missing data on blood lipid profiles (*n* = 6945); (4) excessive alcohol consumption (*n* = 773), defined as consuming 4/5 or more drinks per day (considering the known impact of alcohol on the oral microbiota)^[^22–25^]^; and (5) missing oral microbiome data (*n* = 1484). A total of 3104 participants were ultimately included in the analysis (Fig. 1).

### Definition of hyperlipidemia

Participants with hyperlipidemia were identified according to the guidelines of the National Cholesterol Education Program for adults (2002)^[^26^]^ and met any of the following criteria: (1) TCHO ≥ 200 mg/dL; (2) TG ≥ 150 mg/dL; (3) LDL-C ≥ 130 mg/dL; (4) HDL-C ≤ 40 mg/dL in men or ≤ 50 mg/dL in women; or (5) a previous diagnosis of hyperlipidemia or current use of lipid-lowering medications. The specific procedures for measuring these indicators are outlined on the NHANES Laboratory Methods webpage (https://wwwn.cdc.gov/nchs/data/nhanes/).

### Survival outcomes

Given that hyperlipidemia is a significant risk factor for cardiovascular disease–related mortality^[^27^]^, the primary outcome for survival analysis was cardiovascular disease–related mortality. Cardiovascular disease–related mortality was determined via probabilistic record linkage between NHANES participants and death certificate data from the National Death Index (NDI). This study analyzed NHANES-related NDI public access data up to 31 December 2019.

### Oral microbiome

All participants aged 14–69 years in the 2009–2010 and 2011–2012 surveys were eligible for oral microbiome sampling. The participants were instructed to rinse their mouths with mouthwash for 5 s, after which the mouthwash was expelled into a cup to obtain oral rinse samples. DNA was extracted from oral rinse samples via the PureGene DNA Purification Kit. Polymerase chain reaction amplification was subsequently performed for gene sequencing^[^28^]^. The raw sequencing data were demultiplexed and processed to generate amplicon sequence variants (ASVs)^[^29^]^.

Oral microbial community diversity is represented by alpha diversity indices, which typically reflect community richness and/or evenness^[^30^]^. The alpha diversity metrics included observed ASVs [operational taxonomic units (OTU richness)], phylogenetic diversity [Faith’s phylogenetic diversity (FPD)], and the Shannon‒Weiner index (SWI). These metrics were generated on the basis of rarefaction to 10 000 reads per sample^[^29^]^. OTU richness measures the total number of ASVs observed in a sample; the FPD quantifies richness via phylogenetic tree information; and the SWI assesses both richness and evenness.

Beta diversity, which measures microbiome diversity between individuals, is expressed as pairwise dissimilarities among individuals. In this study, unweighted UniFrac, weighted UniFrac, and Bray‒Curtis dissimilarities between individuals were calculated and represented as distance matrices^[^29^]^.

### Covariates

The following variables were included as covariates in the analysis: age, sex, race (Mexican American, other Hispanic, non-Hispanic White, non-Hispanic Black, other races), marital status (married/living with a partner, never married/widowed/divorced/separated), educational level (less than high school, high school or equivalent, college or higher), poverty-to-income ratio (≤1.0, >1.0), body mass index (BMI), health insurance status, smoking status, physical activity, diabetes, and hypertension. Physical activity was categorized into high and low levels on the basis of whether the participants engaged in moderate-intensity or high-intensity activity. A history of diabetes and hypertension was determined on the basis of questionnaire responses.

### Statistical analysis

Chi-square tests and *t*-tests were used to assess the demographic characteristics of participants stratified by hyperlipidemia status. Spearman’s correlation coefficients were applied to determine correlations between variables. Univariate and multivariate logistic regression analyses were performed to estimate odds ratios (ORs) and 95% confidence intervals (CIs) to investigate the associations between variables and the risk of hyperlipidemia. Univariate and multivariate linear regression analyses were used to explore the relationships between variables and blood lipids. Univariate and multivariate Cox proportional hazards regression analyses were conducted to estimate hazard ratios (HRs) and 95% CIs for examining the associations between variables and cardiovascular disease–related mortality. Subgroup analyses and interaction analyses were performed to explore the interactions between different grouping variables and alpha diversity with respect to mortality risk. Survival curves were plotted via the Kaplan‒Meier method. For the above regression models, Model 1 was unadjusted; the partially adjusted model (Model 2) was adjusted for age, sex, and BMI; and the fully adjusted model (Model 3) was additionally adjusted for race, marital status, physical activity, smoking status, PIR, health insurance status, history of diabetes, and history of hypertension. Missing covariate data were imputed via the multiple imputation by chained equations (MICE) method via the “mice” package^[^31^]^, and a summary of missing covariate information is provided in Supplemental Digital Content Table S1, available at: http://links.lww.com/MS9/B181. Restricted cubic splines (RCSs) were used to investigate nonlinear associations between variables and blood lipid levels.

Principal coordinate analysis (PCoA) and permutational multivariate analysis of variance (PERMANOVA) were used to characterize differences in beta diversity between the hyperlipidemic and non-hyperlipidemic groups. Analyses of the oral microbiota were performed via the “microeco” package^[^32^]^. Linear discriminant analysis effect size (LEfSe) analysis was used to calculate linear discriminant analysis (LDA) scores between different groups, thereby identifying key microbial genera in the hyperlipidemic population^[^33^]^, with an LDA score threshold of 2.0. The functional annotation of prokaryotic taxa (FAPROTAX) was used for functional prediction analysis to explore differences in microbial functions between groups. Mediation analysis was performed to investigate the mediating effect of microbial functions on the association between the oral microbiota and hyperlipidemia.

All analyses were conducted via R (version 4.3.2). A two-tailed significance level of 0.05 was considered statistically significant. Bonferroni correction was applied for multiple testing, with a two-tailed significance level set at 0.017.

## Results

### Descriptive statistics

Of the 3104 participants, 2215 were classified as having hyperlipidemia. As shown in Table 1, the hyperlipidemic group was older (45.01 vs. 35.31, *P* < 0.001), had a higher BMI (29.81 vs. 25.60, *P* < 0.001), and had a higher prevalence of hypertension (32.14% vs. 12.60%, *P* < 0.001) and diabetes (12.78% vs. 4.39%, *P* < 0.001). No significant differences in any of the three alpha diversity indices were observed between the two groups (Supplemental Digital Content Figure S1, available at: http://links.lww.com/MS9/B181).

**Table 1:** Baseline characteristics were based on the presence of hyperlipidemia.

Variables	Total	Hyperlipidemia	Non-hyperlipidemia	*P*
Age	42.31 (0.48)	45.01 (0.50)	35.31 (0.67)	<0.001
Gender				0.240
Male	1410 (45.43)	994 (44.88)	416 (46.79)	
Female	1694 (54.57)	1221 (55.12)	473 (53.21)	
BMI	28.64 (0.20)	29.81 (0.27)	25.60 (0.29)	<0.001
Race				0.212
Mexican American	547 (17.62)	406 (18.33)	141 (15.86)	
Non-Hispanic Black	356 (11.47)	267 (12.05)	89 (10.01)	
Non-Hispanic White	1127 (36.31)	811 (36.61)	316 (35.55)	
Other Hispanic	684 (22.04)	475 (21.44)	209 (23.51)	
Other races	390 (12.56)	256 (11.56)	134 (15.07)	
Education				0.186
Under high school	293 (9.44)	232 (10.47)	61 (6.86)	
High school or equivalent	1016 (32.73)	755 (34.09)	261 (29.36)	
Above high school	1795 (57.83)	1228 (55.44)	567 (63.78)	
Marital				0.003
Married/Living with partner	1807 (58.22)	1350 (60.95)	457 (51.41)	
Widowed/Divorced/Separated/Never married	1297 (41.78)	865 (39.05)	432 (48.59)	
PIR				0.262
Nonpoverty	2352 (75.77)	1674 (75.58)	678 (76.27)	
Poverty	752 (24.23)	541 (24.42)	211 (23.73)	
Insurance				0.152
No	891 (28.70)	623 (28.13)	268 (30.15)	
Yes	2213 (71.30)	1592 (71.87)	621 (69.85)	
Smoke				0.091
No	2011 (64.79)	1388 (62.66)	623 (70.08)	
Yes	1093 (35.21)	827 (37.34)	266 (29.92)	
Hypertension				<0.001
No	2280 (73.45)	1503 (67.86)	777 (87.40)	
Yes	824 (26.55)	712 (32.14)	112 (12.60)	
Diabetes				<0.001
No	2782 (89.63)	1932 (87.22)	850 (95.61)	
Yes	322 (10.37)	283 (12.78)	39 (4.39)	
Physical activity				0.612
High	1087 (35.02)	759 (34.27)	328 (36.90)	
Low	2017 (64.98)	1456 (65.73)	561 (63.10)	
SBP, mmHg	118.58 (0.47)	120.35 (0.51)	114.01 (0.66)	<0.001
DBP, mmHg	70.52 (0.39)	71.57 (0.42)	67.81 (0.52)	<0.001
FPG, mg/dL	102.34 (0.65)	105.10 (0.82)	95.17 (0.51)	<0.001
GHB, %	5.57 (0.02)	5.66 (0.03)	5.32 (0.02)	<0.001
OGTT, mg/dL	121.12 (1.81)	128.83 (2.45)	101.11 (1.80)	<0.001
Glucose, mg/dL	96.46 (0.62)	99.20 (0.78)	89.33 (0.47)	<0.001
Insulin, pmol/mL	81.83 (2.09)	91.19 (2.78)	57.55 (2.07)	<0.001
Albumin, g/dL	4.30 (0.01)	4.28 (0.01)	4.35 (0.02)	0.009
Globulin, g/dL	2.83 (0.02)	2.86 (0.02)	2.77 (0.03)	0.010
Protein, g/dL	7.13 (0.02)	7.13 (0.02)	7.12 (0.03)	0.642
LDL, mg/dL	115.54 (0.69)	123.96 (0.80)	93.71 (0.82)	<0.001
HDL, mg/dL	53.82 (0.47)	51.66 (0.57)	59.41 (0.53)	<0.001
TG, mg/dL	117.44 (2.21)	133.22 (2.62)	76.47 (1.69)	<0.001
TCHO, mg/dL	192.84 (0.93)	202.27 (1.03)	168.40 (0.79)	<0.001
ALT, U/L	25.14 (0.32)	26.60 (0.40)	21.37 (0.45)	<0.001
AST, U/L	25.24 (0.31)	26.05 (0.40)	23.15 (0.32)	<0.001
GGT, IU/L	25.13 (0.78)	27.55 (0.97)	18.85 (0.60)	<0.001

Mean (SE) for continuous variables, *n* (%) for categorical variables.

BMI, body mass index; PIR, poverty-to-income ratio; SBP, systolic blood pressure; DBP, diastolic blood pressure; FPG, fasting plasma glucose; GHB, glycohemoglobin; OGTT, oral glucose tolerance test; LDL, low-density lipoprotein; HDL, high-density lipoprotein; TG, triglycerides; TCHO, total cholesterol; ALT, alanine aminotransferase; AST, aspartate aminotransferase; ALP, alkaline phosphatase; GGT, gamma-glutamyl transferase;

Univariate and multivariate logistic regression analyses were performed to explore the associations between the three alpha diversity indices and the risk of hyperlipidemia (Supplemental Digital Content Table S2, available at: http://links.lww.com/MS9/B181), but no clear associations were found between alpha diversity and hyperlipidemia diagnosis (*P* > 0.017).

### Correlation between alpha diversity and blood lipids

Spearman’s correlation analysis was used to examine the relationships between alpha diversity and the four blood lipid parameters (Supplemental Digital Content Figure S2, available at: http://links.lww.com/MS9/B181). OTU richness (Rho = 0.050, *P* = 0.006), FPD (Rho = 0.060, *P* = 0.001), and SWI (Rho = 0.053, *P* = 0.003) were positively correlated with TG. In contrast, OTU richness (Rho = −0.119, *P* < 0.001), FPD (Rho = −0.118, *P* < 0.001), and SWI (Rho = −0.070, *P* < 0.001) were negatively correlated with HDL. For LDL, only FPD was positively correlated (Rho = 0.044, *P* = 0.015), whereas no significant correlations were detected for OTU richness or SWI. None of the three alpha diversity indices were significantly correlated with TCHO.

Furthermore, multivariate linear regression analyses were conducted to further investigate the associations between alpha diversity and blood lipids (Table 2). In the fully adjusted model, only the positive correlation between FPD and TG remained significant (beta = 1.028, 95% CI: 0.070–1.985, *P* = 0.037), but this significance was lost after Bonferroni correction. All three alpha diversity indices showed significant positive correlations with TCHO and LDL in the fully adjusted model (*P* < 0.017), whereas a significant correlation with HDL was no longer observed (*P* > 0.05).

**Table 2:** Linear model for blood lipid biomarkers.

Outcomes	Variables	Model 1		Model 2		Model 3	
		Beta (95% CI)	*P*	Beta (95% CI)	*P*	Beta (95% CI)	*P*
TG	OTU_richness	0.062 (−0.008 to 0.133)	0.082	0.082 (0.006–0.157)	0.034	0.060 (−0.021 to 0.141)	0.139
	FPD	0.996 (0.104-1.888)	0.030	1.274 (0.376–2.172)	0.007	1.028 (0.070–1.985)	0.037
	SWI	4.122 (−0.185 to 8.430)	0.060	4.565 (0.126–9.003)	0.044	3.341 (−1.210 to 7.892)	0.140
TCHO	OTU_richness	−0.010 (−0.052 to 0.033)	0.653	0.058 (0.016–0.100)	0.008	0.055 (0.017–0.093)	0.007
	FPD	0.020 (−0.511 to 0.550)	0.940	0.888 (0.364–1.413)	0.002	0.871 (0.402–1.340)	0.001
	SWI	1.542 (−0.979 to 4.063)	0.222	4.472 (2.105-6.839)	0.001	4.461 (2.322–6.600)	<0.001
HDL	OTU_richness	−0.042 (−0.061 to 0.022)	<0.001	−0.017 (−0.035 to 0.000)	0.053	−0.010 (−0.028 to 0.007)	0.240
	FPD	−0.529 (−0.777 to 0.281)	<0.001	−0.224 (−0.454 to 0.005)	0.055	−0.127 (−0.351 to 0.097)	0.250
	SWI	−1.778 (−3.314 to 0.241)	0.025	−0.576 (−1.783 to 0.631)	0.337	−0.248 (−1.505 to 1.009)	0.683
LDL	OTU_richness	0.020 (−0.015 to 0.055)	0.251	0.060 (0.026–0.093)	0.001	0.053 (0.023–0.084)	0.002
	FPD	0.353 (−0.088 to 0.794)	0.113	0.861 (0.442–1.281)	<0.001	0.797 (0.424–1.169)	<0.001
	SWI	2.501 (0.200 to 4.801)	0.034	4.140 (1.884–6.396)	0.001	4.046 (2.012–6.080)	0.001

Model 1 was unadjusted.

Model 2 adjusted for age, gender, and BMI.

Model 3 additionally adjusted for race, marital status, education, PIR, insurance, smoking, physical activity, hypertension, and diabetes.

**Figure 2.**
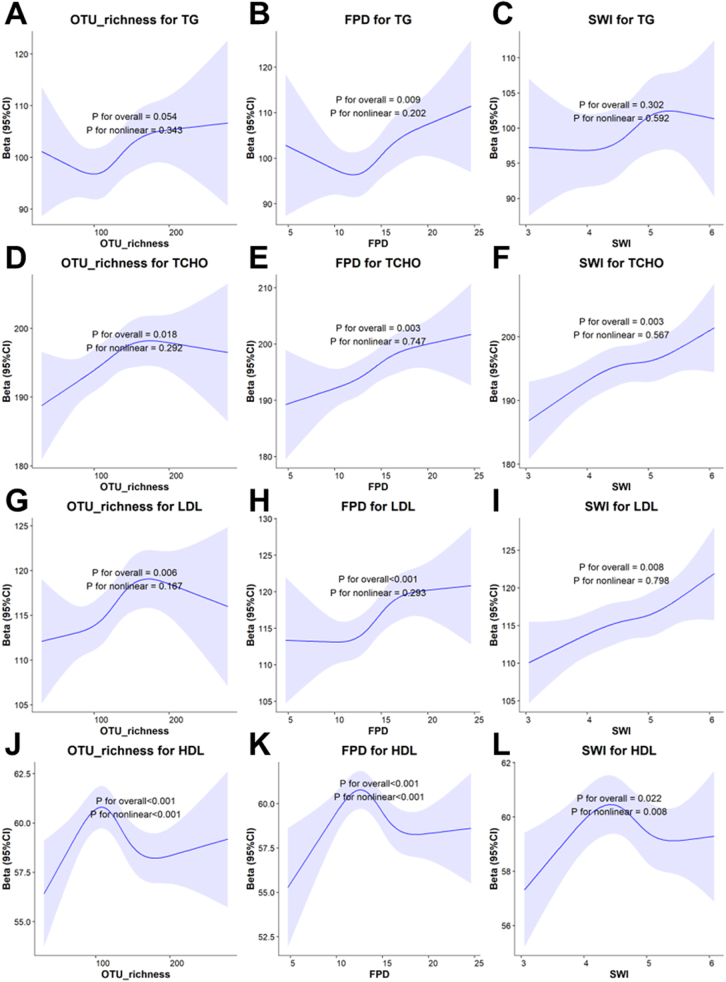
RCSs for the nonlinear associations between alpha diversity and four blood lipids. (A‒C): Effects of OTU richness (A), FPD (B), and SWI (C) on TG. (D–F): Effects of OTU richness (D), FPD (E), and SWI (F) on TCHO. (G–I): Effects of OTU richness (G), FPD (H), and SWI (I) on LDL. (J‒L) Effects of OTU richness (J), FPD (K), and SWI (L) on HDL.

HR, hazard ratio; CI, confidence interval; BMI, body mass index; PIR, poverty-to-income ratio; LDL, low-density lipoprotein; HDL, high-density lipoprotein; TG, triglycerides; TCHO, total cholesterol; OTU, operational taxonomic units; FPD, Faith’s phylogenetic diversity; SWI, Shannon‒Wiener index;

Multivariate-adjusted RCS was also used to explore nonlinear associations between the three alpha diversity indices and the four blood lipids (Fig. 2). OTU richness (Fig. 2J), FPD (Fig. 2K), and SWI (Fig. 2L) all exhibited inverted U-shaped nonlinear associations with HDL-C (*P* for nonlinearity < 0.017). No significant nonlinear associations were found between the three alpha diversity indices and TG (Fig. 2A–C), TCHO (Fig. 2D–F), or LDL (Fig. 2G–I) (P for nonlinearity > 0.017).

### Association between alpha diversity and risk of cardiovascular-related mortality

The association between oral microbiota alpha diversity and cardiovascular disease-related mortality was examined via survival analyses (Table 3). Kaplan‒Meier analysis (Supplemental Digital Content Figure S3, available at: http://links.lww.com/MS9/B181) revealed that participants with higher alpha diversity had a lower risk of cardiovascular mortality (*P* < 0.001). However, in the fully adjusted Cox regression model (Table 3), no significant association was observed between alpha diversity and cardiovascular disease-related mortality risk (*P* > 0.05).

**Table 3:** Cox regression analysis for investigating the correlation between alpha diversity and cardiovascular disease–related mortality.

Variables	Model 1		Model 2		Model 3	
	HR (95% CI)	*P*	aHR (95% CI)	*P*	aHR (95% CI)	*P*
OTU_richness	0.991 (0.983–1.000)	0.038	0.996 (0.987–1.004)	0.311	0.997 (0.988–1.005)	0.433
FPD	0.912 (0.821–1.013)	0.087	0.965 (0.867–1.074)	0.516	0.977 (0.881–1.083)	0.660
SWI	0.671 (0.420–1.072)	0.095	0.786 (0.477–1.297)	0.346	0.809 (0.489–1.341)	0.411

Model 1 was unadjusted.

Model 2 adjusted for age, gender, and BMI.

Model 3 additionally adjusted for race, marital status, education, PIR, insurance, smoking, physical activity, hypertension, and diabetes.

BMI, body mass index; CI, confidence interval; FPD, Faith’s phylogenetic diversity; HR, hazard ratio; OTU, operational taxonomic units; PIR, poverty-to-income ratio; SWI, Shannon–Weiner index.

In subgroup analyses (Supplemental Digital Content Table S3, available at: http://links.lww.com/MS9/B181), statistically significant associations with cardiovascular-related mortality risk were observed for OTU richness (*P* = 0.006), FPD (*P* = 0.013), and SWI (*P* = 0.027) in the smoker subgroup. Additionally, interactions were detected between smoking status and both OTU richness (*P* for interaction = 0.029) and FPD (*P* for interaction = 0.044).

### Microbiome characteristics of the hyperlipidemic population

**Figure 3.**
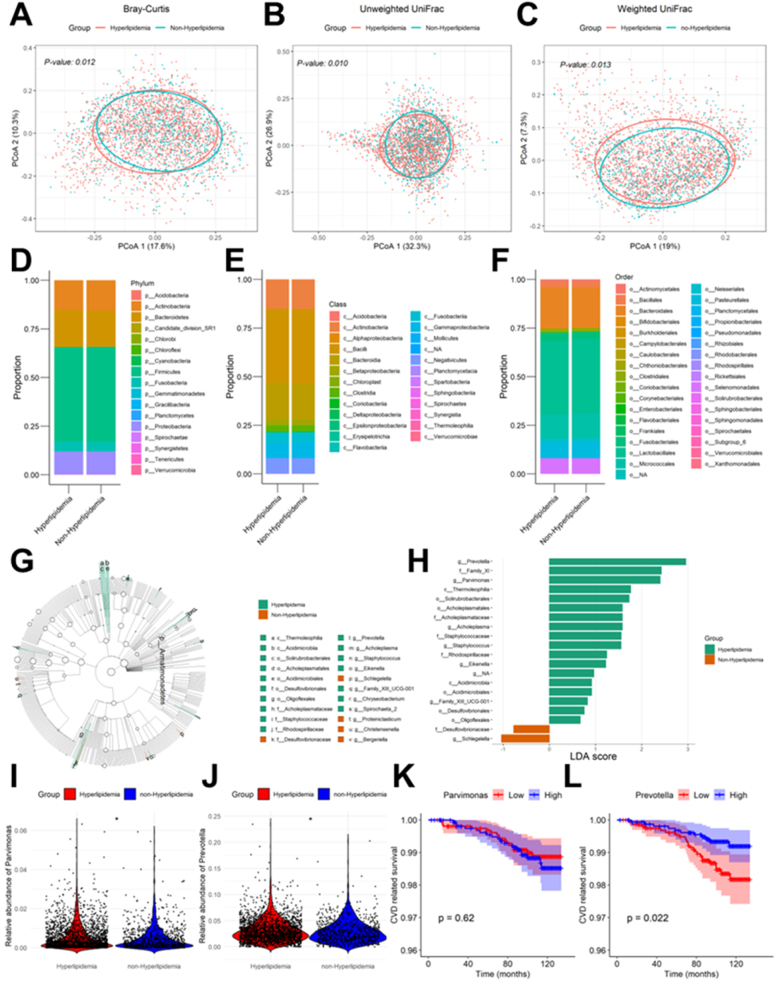
Comparison of the oral microbiome between the hyperlipidemia group and the non-hyperlipidemia group. (A‒C): PCoA of beta diversity for Bray‒Curtis dissimilarity (A), unweighted UniFrac dissimilarity (B), and weighted UniFrac dissimilarity (C). (D–F): Taxa stacked bar plot between groups at the phylum (D), class (E) and order (F) levels. (G) Cladogram showing the group with a significant difference in abundance. (H) Linear discriminant analysis (LDA) was used to identify the significantly abundant genera in the different groups. (I‒J): Comparison of the relative abundances of *Prevotella* (I) and *Parvimonas* (J) between the groups. (K‒L): Kaplan‒Meier results of cardiovascular disease-related mortality according to *Prevotella* (K) and *Parvimonas* (L) abundance.

PERMANOVA tests for beta diversity revealed significant differences in the Bray‒Curtis distance (*P* = 0.012), unweighted UniFrac distance (*P* = 0.010), and weighted UniFrac distance (*P* = 0.013) between the hyperlipidemic and non-hyperlipidemic groups, indicating distinct microbial community compositions between the two groups (Fig. 3A–C).

Stacked bar charts illustrating the microbial distribution at the phylum (Fig. 3D), class (Fig. 3E), and order (Fig. 3F) levels revealed no obvious differences between the groups. LEfSe analysis was further performed to identify differential taxa at the genus level (Fig. 3G–H). Cladogram analysis revealed limited differential genera between the groups (Fig. 3G). LDA revealed that only *Prevotella* and *Parvimonas* had |LDA scores| > 2.0 at the genus level; thus, these genera were identified as key genera in the oral microbiota of hyperlipidemic individuals (Fig. 3H).

Violin plots further confirmed that the abundances of *Prevotella* (Fig. 3I) and *Parvimonas* (Fig. 3J) were greater in the hyperlipidemic group. Additionally, Kaplan‒Meier survival analysis revealed that increased *Prevotella* (Fig. 3K) abundance was associated with a lower risk of cardiovascular disease–related mortality (*P* = 0.022), whereas no significant association was detected between *Parvimonas* (Fig. 3L) abundance and cardiovascular mortality risk (*P* = 0.620).

### *Correlations of* Prevotella *and* Parvimonas *with hyperlipidemia*

**Figure 4.**
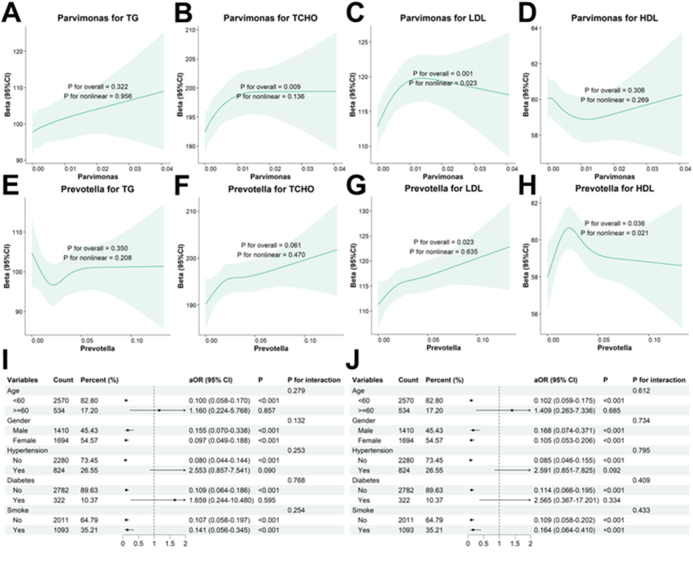
Associations between *Parvimonas*/*Prevotella* and hyperlipidemia. (A-D): RCS for the nonlinear associations of Parvimonas with TG (A), TCHO (B), LDL (C), and HDL (D) levels. (E–H) RCS for the nonlinear associations of Parvimonas with TG (E), TCHO (F), LDL (G), and HDL (H) levels. (I-J): Subgroup analyses for the associations between *Parvimonas* (I)/*Prevotella* (J) and hyperlipidemia.

Spearman’s correlation analysis was performed to examine the linear relationships between *Parvimonas* (Supplemental Digital Content Figure S4 A–D, available at: http://links.lww.com/MS9/B181)/*Prevotella* (Supplemental Digital Content Figure S4 E–H, available at: http://links.lww.com/MS9/B181) and blood lipids. *Parvimonas* was significantly correlated with all four blood lipid parameters, whereas *Prevotella* was only weakly negatively correlated with LDL (*P* = 0.041). Fully adjusted RCS analyses were also conducted to explore nonlinear associations between *Parvimonas* (Fig. 4A–D)/*Prevotella* (Fig. 4E–H) and blood lipids. The results revealed an inverted L-shaped nonlinear association between *Parvimonas* and LDL (*P* for nonlinearity = 0.023) and an inverted U-shaped nonlinear association between *Prevotella* and HDL (*P* for nonlinearity = 0.021).

Subgroup analyses via logistic regression revealed that, despite the absence of interaction effects, the associations of *Parvimonas* (Fig. 4I) and *Prevotella* (Fig. 4J) with hyperlipidemia were not statistically significant in elderly participants, hypertensive participants, or diabetic participants (*P* > 0.05).

### FAPROTAX functional prediction analysis

**Figure 5.**
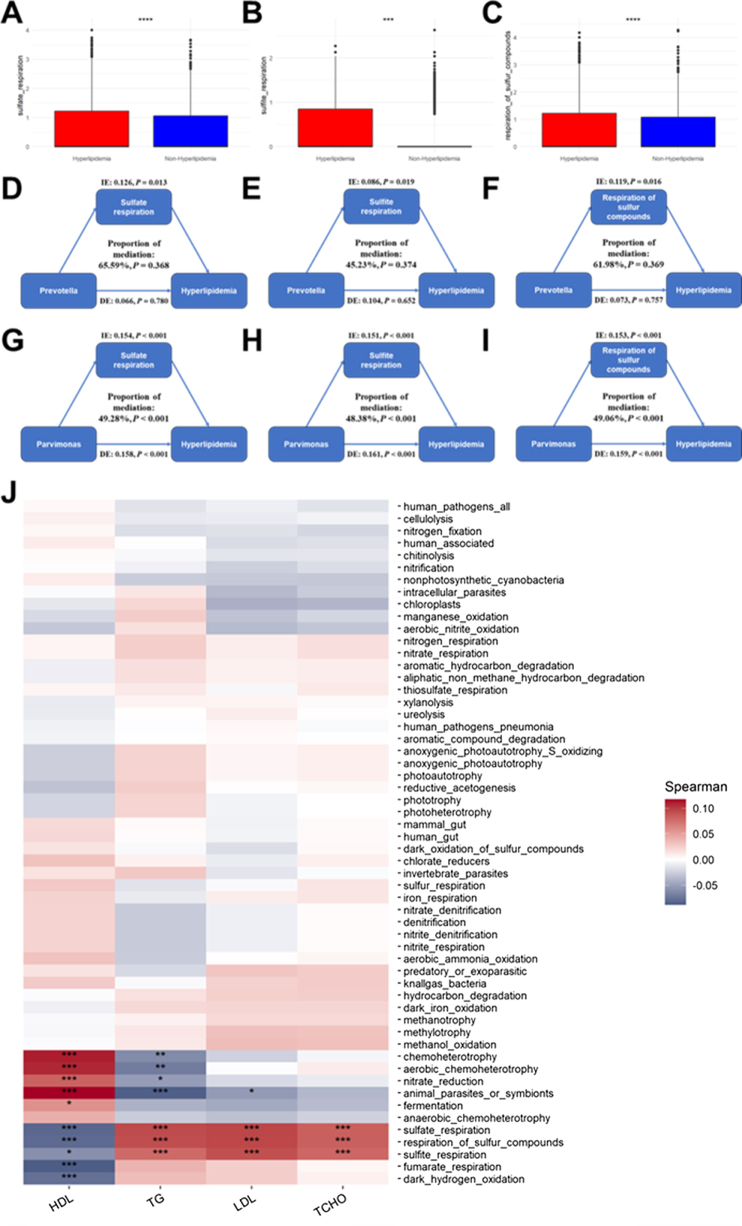
FAPROTAX analysis for the association between microbial function and hyperlipidemia. (A–C): Predicted abundance of the respiration of sulfur compounds (A), sulfate respiration (B), and sulfite respiration (C) between the groups. (D‒F) Estimated proportions of the associations between *Parvimonas* and hyperlipidemia mediated by sulfate respiration (D), sulfite respiration (E), and the respiration of sulfur compounds (F). (G) Correlation heatmap of the associations between microbial function and four blood lipids.

To explore functional differences in microbial communities between groups, FAPROTAX analysis was performed (Supplemental Digital Content Table S4, available at: http://links.lww.com/MS9/B181). In the hyperlipidemic group, relatively high abundances of respiratory processes involving sulfur compounds (Fig. 5A), sulfate respiration (Fig. 5B), sulfite respiration (Fig. 5C), and chlorate reduction (Supplemental Digital Content Figure S5, available at: http://links.lww.com/MS9/B181) were detected.

Mediation analyses (Fig. 5D–F) indicated that *Parvimonas* may increase the risk of hyperlipidemia through sulfate respiration (mediation proportion: 49.28%, *P* < 0.001), sulfite respiration (mediation proportion: 48.38%, *P* < 0.001), and sulfur compound respiration (mediation proportion: 49.06%, *P* < 0.001). No significant mediation effects were observed for *Prevotella* in the mediation analyses (Supplemental Digital Content Figure S6 A–C, available at: http://links.lww.com/MS9/B181).

Additionally, a Spearman correlation heatmap (Fig. 5G) demonstrated correlations between 56 microbial functions and the four blood lipids, revealing significant associations between sulfur compound metabolism and all four blood lipids.

## Discussion

Hyperlipidemia is a microbiome-associated disease^[^14^]^. To our knowledge, this is the first study to systematically analyze the associations between the oral microbiota and host lipid metabolism in a large national cohort. These results indicate that the oral microbiome differs between individuals with hyperlipidemia and healthy controls, providing a theoretical basis for managing hyperlipidemia through oral microbiota modulation.

The alpha diversity of the oral microbiota has been linked to the risk of COPD, depression, and cardiovascular mortality^[^19,34,35^]^. In our study, no significant difference in alpha diversity was detected between the hyperlipidemic and non-hyperlipidemic groups. However, correlation analyses with blood lipids revealed positive associations between alpha diversity and TCHO and LDL, as well as an inverted U-shaped nonlinear association with HDL. These findings suggest that the introduction of new species into the oral cavity may alter host lipid metabolism, potentially contributing to the risk of hyperlipidemia.

Previous studies have reported associations between oral microbiota alpha diversity and all-cause or cardiovascular mortality^[^34,36–38^]^, and our findings validate these results in a subset of the population. Additionally, subgroup analyses suggested stronger associations in smokers and individuals with hyperlipidemia. However, no significant association between alpha diversity and cardiovascular mortality was observed in the fully adjusted Cox regression model, possibly because of insufficient statistical power resulting from a small number of cardiovascular death events. In subgroup analyses, higher alpha diversity was associated with a lower risk of cardiovascular-related death only in smokers, with interactions detected between smoking status and OTU richness/FPD regarding cardiovascular mortality risk. Smoking is known to disrupt the homeostasis of the oral microbiota^[^39,40^]^, suggesting that alpha diversity could serve as a biomarker for assessing the adverse effects of smoking.

LEfSe analysis identified *Parvimonas* (LDA score = 2.41) and *Prevotella* (LDA score = 2.96) as key genera in the oral microbiota of individuals with hyperlipidemia. A small-scale study of 39 participants by Khocht *et al* revealed that oral *Parvimonas* and *Prevotella* were associated with obesity^[^41^]^, whereas another study of 166 obese patients with periodontitis reported increased proportions of these genera^[^42^]^. Our study confirms the association between these genera and hyperlipidemia in a larger cohort. In subgroup analyses, despite no detected interactions, the associations of *Parvimonas* and *Prevotella* with hyperlipidemia lost statistical significance in elderly individuals or those with hypertension or diabetes. This may be attributed to the significant overlap between these populations and individuals with hyperlipidemia.

The relationship between *Prevotella* and lipid metabolism remains controversial. In studies on the gut microbiota, *Prevotella copri* has been positively associated with obese phenotypes^[^43^]^. The protective effect of the Mediterranean diet against cardiometabolic diseases is more pronounced in individuals with lower *Prevotella copri* abundance^[^44^]^. In animal experiments, *Prevotella copri* increased fat accumulation in pigs^[^45^]^. Some studies have shown that interventions targeting hyperlipidemia reduce the abundance of gut *Prevotella*^[^46,47^]^. However, other studies reported lower *Prevotella* abundance in hyperlipidemic states^[^48,49^]^. Importantly, previous studies focused on intestinal *Prevotella*, whereas our work investigated the association between oral *Prevotella* and lipid metabolism.

*Parvimonas parva* was not identified until 2021^[^50^]^, whereas *Parvimonas micra* is a well-known species within the *Parvimonas* genus and represents the primary species of this genus. *Parvimonas micra* is recognized as a bacterium with adverse effects on the host and acts as a key proinflammatory oral pathogen^[^51,52^]^. Its presence increases the risk of oral cancer and colorectal cancer^[^53–56^]^. Additionally, *Parvimonas micra* has been linked to the development of atherosclerosis^[^57^]^.

Functional prediction and mediation analyses revealed that sulfur compound respiration is significantly associated with host lipid metabolism and that *Parvimonas* may increase hyperlipidemia risk through sulfur compound respiration. A key product of sulfur compound respiration is hydrogen sulfide^[^58^]^, and *Parvimonas* has been identified as a major genus involved in hydrogen sulfide production in the oral cavity^[^59^]^. Exogenous hydrogen sulfide promotes adipocyte differentiation, proliferation, and hypertrophy^[^60^]^. We hypothesize that *Parvimonas* may contribute to hyperlipidemia through hydrogen sulfide production. However, due to the predictive and inferential nature of FAPROTAX analysis, these findings remain at the computational level and lack direct experimental evidence, which should be interpreted with caution. The ultimate goal of our research was to regulate lipid metabolism through oral microbiota management, highlighting the need for further studies to clarify whether oral microbial sulfur metabolism increases the risk of hyperlipidemia.

From a clinical perspective, our findings suggest that the oral microbiome may serve as a noninvasive biomarker for screening populations at high risk of hyperlipidemia. Targeted regulation of the oral microbiota, such as controlling the abundance of *Parvimonas*, reducing sulfur metabolism-related pathways, and maintaining oral microbial homeostasis, may provide new adjuvant strategies for the prevention and management of hyperlipidemia. Furthermore, artificial intelligence (AI) technology and large language models have shown revolutionary potential in medical research and multi-omics data integration^[^61,62^]^. Combining oral microbiome data with advanced AI models will help to develop more accurate risk prediction models for hyperlipidemia and support the clinical translation of microbiome-targeted therapies.

This study has several limitations. The NHANES database does not provide species-level microbiome data, limiting resolution when identifying key oral microbial species. The cross-sectional design precludes definitive causal inferences; hyperlipidemia itself may increase the colonization of some pathogenic microbes in the oral cavity rather than the reverse^[^63^]^. The single-country cohort restricts the generalizability of the results. Although we attempted to correlate hyperlipidemia-related data with matched cardiovascular survival outcomes, the low incidence of events necessitates cautious interpretation. Despite systematic correlation analyses between the oral microbiota and hyperlipidemia, this study lacked experimental validation of the findings, which will be a focus of future research.

## Conclusion

In summary, this study is the first to systematically investigate the association between the oral microbiome and hyperlipidemia in a large national cohort. The alpha diversity of the oral microbiota is positively correlated with blood LDL and TCHO levels and has an inverted U-shaped nonlinear association with blood HDL levels. *Parvimonas* and *Prevotella* are characteristic genera in the oral microbiota of individuals with hyperlipidemia. *Parvimonas* may increase the risk of hyperlipidemia through sulfur metabolism. Further mechanistic studies are needed to validate these findings and clarify the role of the oral microbiota in the pathogenesis of hyperlipidemia.

## Data Availability

The datasets used for all analyses in this research are publicly available on the NHANES website (https://www.cdc.gov/nchs/nhanes/).
